# Magnetic heterogeneous catalytic ozonation: a new removal method for phenol in industrial wastewater

**DOI:** 10.1186/2052-336X-12-50

**Published:** 2014-02-26

**Authors:** Yousef Dadban Shahamat, Mahdi Farzadkia, Simin Nasseri, Amir Hossein Mahvi, Mitra Gholami, Ali Esrafili

**Affiliations:** 1 Department of Environmental Health Engineering, School of Public Health, Iran University of Medical Sciences, Tehran, Iran; 2 Center for Water Quality Research (CSWR), Institute for Environmental Research and Department of Environmental Health Engineering, School of Public Health, Tehran University of Medical Sciences, Tehran, Iran

**Keywords:** Catalytic ozonation, Magnetic nano-composite, Phenol, Sequencing batch reactor

## Abstract

In this study, a new strategy in catalytic ozonation removal method for degradation of phenol from industrial wastewater was investigated. Magnetic carbon nano composite as a novel catalyst was synthesized, characterized and then used in the catalytic ozonation process (COP) and compared with the single ozonation process (SOP). The influential parameters were all investigated. The results showed that the removal efficiency of phenol and COD (chemical oxygen demand) in COP (98.5%, 69.8%) was higher than those of SOP (78.7%, 50.5%) and the highest catalytic potential was achieved at optimal neutral pH. First order modeling demonstrated that the reactions were dependent on the concentration of catalyst, with kinetic constants varying from 0.023 1/min (catalyst = 0 g/L) to 0.071 1/min (catalyst = 4 g/L), whereby the optimum dosage of catalyst was found to be 2 g/L. Furthermore, the catalytic properties of the catalyst remained almost unchanged after 5-time reuse. The results regarding the biodegradability of the effluent showed that a 5-min reaction time in COP reduced the concentrations of phenol and COD to the acceptable levels for the efficient post-treatment in the SBR in a 4-h cycle period. Finally, this combined system is proven to be a technically effective method for treating phenolic contaminants.

## Introduction

Ozonation is one of the oxidation processes widely used for industrial wastewater pretreatment in which ozone molecules (as a strong oxidant) break down recalcitrant and toxic organic compounds into smaller molecules. The ozonation reaction is accomplished through two pathways: direct ozone oxidation and indirect free hydroxyl radical oxidation.

In direct ozonation, organic molecules can be destroyed in various ways, including: **a)** the breakage of double bond and formation of aldehydes and ketones, **b)** the addition of an oxygen atom to the benzene ring and **c)** the reaction with alcohols to form organic acids [1]. In indirect free hydroxyl radical oxidation ozone is decomposed to free reactive radicals, which can cause a significant rise in pollutant removal efficiency [2,3]. However, ozonation has some limitations such as: (1) high energy consumption for ozone generation which could be costly; (2) in some cases ozonation is selective; (3) incomplete oxidation and low efficiency due to low reaction kinetics and limited mass transfer; [4] and incomplete mineralization of recalcitrant organics [5]. For instance, when ozonation is used alone, saturated intermediates may accumulate in the effluent solution which are more toxic than initial pollutant, especially at the early stage of ozonation [4]. Thus, studies were focused on improving the ozonation efficiency and overcoming the weakness of the single ozonation process (SOP).

Many studies have reported that the mineralization of different toxic and bio recalcitrant of organic compounds can be improved by combining ozonation with other agents such as UV, H_2_O_2_ and homogeneous catalytic ozonation; for instance, MnO_2_, Mn^+2^, Co_3_O_4_, RuO_2_, Fe^+3^, Fe^+2^, Ag^+^, Zn^+2^,Ru, MgO, Ni, Cu, and Co^+2^[2,6] coupled with advanced oxidation processes (AOPs). But, due to high consumption of the catalyst and complexity of these technologies, it is rarely selected as a promising method.

Recently, heterogeneous catalytic ozonation processes (COP), as a powerful treatment method, have been investigated to increase the efficiency of ozonation process. In this process, a synthesized catalyst is applied to increase the ozone decomposition and thereby forming highly reactive free radicals. Many catalysts including metals, metal oxides such as CO_3_O_4_/CeO_2_, TiO_2_ Pt/Carbon nanotube (CNT), Ru/Al_2_O_3_, Mn/TiO_2_, Au/AC, Mn/Co, Fe_3_O_4_/CoO, ZnO, Fe_2_O_3_, Fe_2_O_3_/CeO_2_, CNT, Ru/CeO_2_ Cu/ZrO CuFe_2_O_4_ and activated carbon (AC) are widely used for enhancing the activation of the ozonation process [7-10]. Nevertheless, the main limitations of these catalysts are the complexity and high cost of synthesis, and the leaching of materials into the liquid system causing high consumption of catalyst and being regarded as a new pollutant in a treated wastewater. Hence, this research is focusing on a new material with a high catalytic and reusable properties and it should also be easy and cheap to produce. In recent studies, the coupling of Ozone and activated carbon was proven to be an effective method to degrade organic contaminants [11,12]. In this method, carbon can act as an adsorbent, a reactive support and free-radical initiator. These free-radicals can degrade a wide variety of organic contaminants in wastewater [4,9], but irreversibility and high consumption still remain the main disadvantages of this catalyst.

In this study, carbon nano-composite was applied as a catalyst for heterogeneous ozonation and also applied for the determination of the potential of catalytic activity. This nano-composite has superparamagnetic properties that could be recovered from effluent by magnetic field and reused for several times even without deactivation of catalyst.

Since phenol is a toxic and hazardous contaminant [13-15], it is selected as a model of toxic organic contaminant in COP. Finally, the catalytic activity of the catalyst and the influence of some operational conditions such as initial pH of the solution, the amounts of catalyst and the reaction time on the phenol degradation and the mineralization efficiencies were evaluated.

For control and optimization of this treatment method, it is necessary to understand the role of this catalyst and the nature of the reactions. Accordingly, in this research, the physical and chemical properties of the nano-composite, such as specific surface area, pHzpc and its composition were determined and later discussed in detail. In order to explain the nature of reactions, the experiments were performed using tert-butanol as radical scavenger. Also, the degradation kinetics of phenol was determined and discussed via SOP and COP experiments.

The alternative method which was used in most of the previous studies of AOPs [16-18] is the partial oxidation of toxic contaminant in COP followed by further treatment in a biological process. Since the majority of AOPs, for complete treatment and economic considerations, are associated with aerobic biological treatment, the mineralization and biodegradability of pretreated phenol with COP at the optimum conditions were analyzed by a bench scale sequencing batch reactor (SBR) reactor. The aim of the analysis was to evaluate the efficiency of degradation and COD removal of phenol in the combined process.

## Materials and methods

### Chemicals

Phenol (purity ≥ 99.5%; CAS No.: 108-95-2) was purchased from Merck Co. (Germany). Then standard solutions were prepared with distilled and deionized water and protected from light, and stored at 4°C. Except for HPLC-grade acetonitrile, all other chemical agents such as (KI), (Na_2_S_2_O_5_), (Na_2_SO_3_), sulfuric acid, nitric acid, Sodium Hydroxide, (KCr_2_O_7_), (Ag_2_SO_4_), (HgSO_4_), NaH_2_PO_4_, tert-butyl alcohol and Fe (NO3)3 · 9H2O were of analytical reagent grade.

A commercial powder activated carbon (CAS No.: 1.02183.1000) supplied by Merck Co. was used in this study as a precursor of the catalyst, which its characterization compared with the final catalyst shown in Table 1.

**Table 1:** **Specifications of nano- Fe**_**3**_**O**_**4 **_**coated on activated carbon**

**Parameters**	**PAC**	**Fe**_**3**_**O**_**4**_**/AC**
Specific surface area (m^2^/g)	907	814
Pore volume (cm^3^/g)	0.42	0.26
pHzpc	8.9	7.7
Average particle size as Fe_3_O_4_	-	25-30
Assay (%) as carbon	99.99	89.2
Color	Black	Black

### Preparation and characterization of the catalyst

The nano-composite catalyst was prepared from activated carbon (AC) via a modified impregnation method by using Fe_3_O_4_. Firstly, the AC was treated with nitric acid (37%) and the resulting mixture was kept at 80°C and stirred for 3 h to make it hydrophilic. Then, it was washed with water, filtered and dried at 105°C over night. 25 g of the modified AC was dispersed in 200 ml aqueous solution containing 100 g Fe (NO3)3 · 9H2O as a Fe_3_O_4_ precursor by sonication using an ultrasonic bath. The resulting particles then filtrated, dried and the thermal treatment was performed at 700°C for 1 h in the presence of pure nitrogen flow for the formation of Fe3O4 magnetic nano particles [19].

The specific surface area of the prepared catalyst was determined using the BET equation and its mineralogical characterization was specified by X-ray diffraction (XRD) patterns carried out on an XRD difractometer at room temperature. The distribution of elements on the catalyst was determined by Dispersive X-ray Spectroscopy (EDS) and its morphology was characterized by Scanning Electron Microscopy (SEM) analysis. The pH of the zero point of charge (pHzpc) of the catalyst was measured by acid-base titration of catalyst suspension method, which is detailed by Altenor et al. [20].

### Catalytic ozonation treatment

The ozone was generated from pure oxygen via corona discharge using an ozone generator (ARDA, Model COG-1A) with 5 g O_3_/h capacity. The ozone inlet flow rate was controlled via a gas rotameter (capacity, 3.5 L/min) at 0.5 L/min.

The ozone was regulated at a constant mass flow rate of 33 mg/(L.min) throughout the experiments measured by the standard potassium iodide (KI) absorption method [21], and finally destroyed in the off-gas stream of the reactor in a concentrated KI solution.

The samples containing phenol were prepared from the stock solution (5000 mg/L) and their residual concentrations in the samples were analyzed by HPLC (Cecil CE 4100) using a Hypersil C18 column (250 mm × 4.6 mm i.d, with 5 μm particle size) with a UV detector (Cecil CE 4200) at 254 nm. The Mobile phase consisted of a mixture of 50 mM buffer solution (NaH_2_PO_4_) and acetonitrile (50:50, v: v) at a flow rate of 1.0 mL/min.

The experiments regarding the catalytic ozonation were carried out in a semi batch cylindrical stainless steel reactor with 1.9 L total volume (diameter = 5 cm and height = 100 cm) fitted with other elements including an ozone generator, a sintered diffuser to distribute the ozone air stream into the solution, a cylinder of pure oxygen (99.9%), an ozone off-gas trap system and gas rotameter. For each catalytic ozonation test the following procedure was made:

1- A 1 L solution of phenol with a certain initial concentration was produced in a 2 L glass beaker.

2- The initial pH of the solution was adjusted at the desired value by the addition of either NaOH or HCl (1 M).

3- The required amount of catalyst was added to the solution.

4- The solution was transferred into the reactor and the ozonation was started at a certain time (See Table 2).

**Table 2:** Experimental steps and conditions

**Phase**	**Experiment**	**Conditions**			
		**C**_**Phenol **_**(mg/L)**	**C**_**Catalyst **_**(g/L)**	**pH**	**Time (min)**
1	Effect of pH	100	0.5 ^a^	4-10	5
2	Effect of catalyst concentration loading	500	0.2-4^a^	8^b^	0-60
3	Effect of radical scavenger	500	2^a^	8	0-60
4	Adsorption, aeration and synergistic effect	500	2	8	0-60
5	Phenol and COD removal at optimum condition	500	2	8	0-60
6	Biodegradability of the COP effluent	500	2	8	5
7	Catalyst durability	500	2	8	60

^a^The same experiments were carried out for SOP (without catalyst).

^b^Optimum pH in which the maximum phenol removal was obtained in COP.

At given time intervals, 2 mL of the sample was immediately introduced into 100 μL of sulphite solution (0.1 M) to remove the dissolved ozone [22]. The magnet and filter (0.22-μm) were used to remove the catalyst and then 60 μL of the recovered sample was injected to the HPLC for analysis of the residual phenol. The experimental runs are based on one factor at a time approach, which conditions are detailed in Table 2.

At the optimum condition, the extent of mineralization of the organic matter was measured by COD experiment via digestion of the pre-treated sample with KCr_2_O_7_ solution [21].

### Kinetic studies

The kinetic study in both COP and SOP was carried out with different concentrations of catalyst and phenol. The following first-order kinetic expression was used to determine the phenol removal reaction rate, given by Eq. 1:

(1) $$\ln\;\left(C/C_{0}\right)=\mathrm{kt}$$

Where, k is the first-order rate constant, C and C0 are the phenol concentrations at reaction time t and t_0_, respectively.

### Biodegradability of COP effluent

For mineralization of the COP effluent, a series of biological treatments were carried out on pre-treated sample of the COP effluent resulting from the optimum conditions shown in phase 6 of Table 2. The SBR reactor used in this study was a 1-liter glass graduated cylinder with a total effective volume of 0.5 L; and in each test of this stage, 375 mL of solution of COP effluent was poured into a biological reactor for aeration. Then, 125 mL of acclimated and condensed activated sludge (1.4% solids) was added to the reactor and the system was operated in a 4-h cycle period (2 min filling, 233 min reacting, and 5 min settling and decanting). After the prescribed reaction time, the mixed liquor was settled and analyzed for residual phenol and COD concentration. Since the effluent was decanted at 250 mL of effective volume of reactor (500 mL), Hydraulic Retention Time (HRT), was calculated 8 h. The parameter of sludge retention time (SRT) in the SBR depends on sludge settling properties and was not controlled during this study.

## Results and discussion

### Characterization of catalyst particles

The surface area and the total pore volume (at P/P_o_ = 0.992) of the catalyst were 814 m^2^/g and 0.26 cm^3^/g, respectively. The surface area of magnetic catalyst was reduced from 907 m^2^/g to 814 m^2^/g (10% reduction), attributing to the formation of nano-particles of Fe_3_O_4_ inside the pores. Nano-particles of Fe_3_O_4_ bonded on the surface of activated carbon by hydroxyl groups [23].

**Figure 1:**
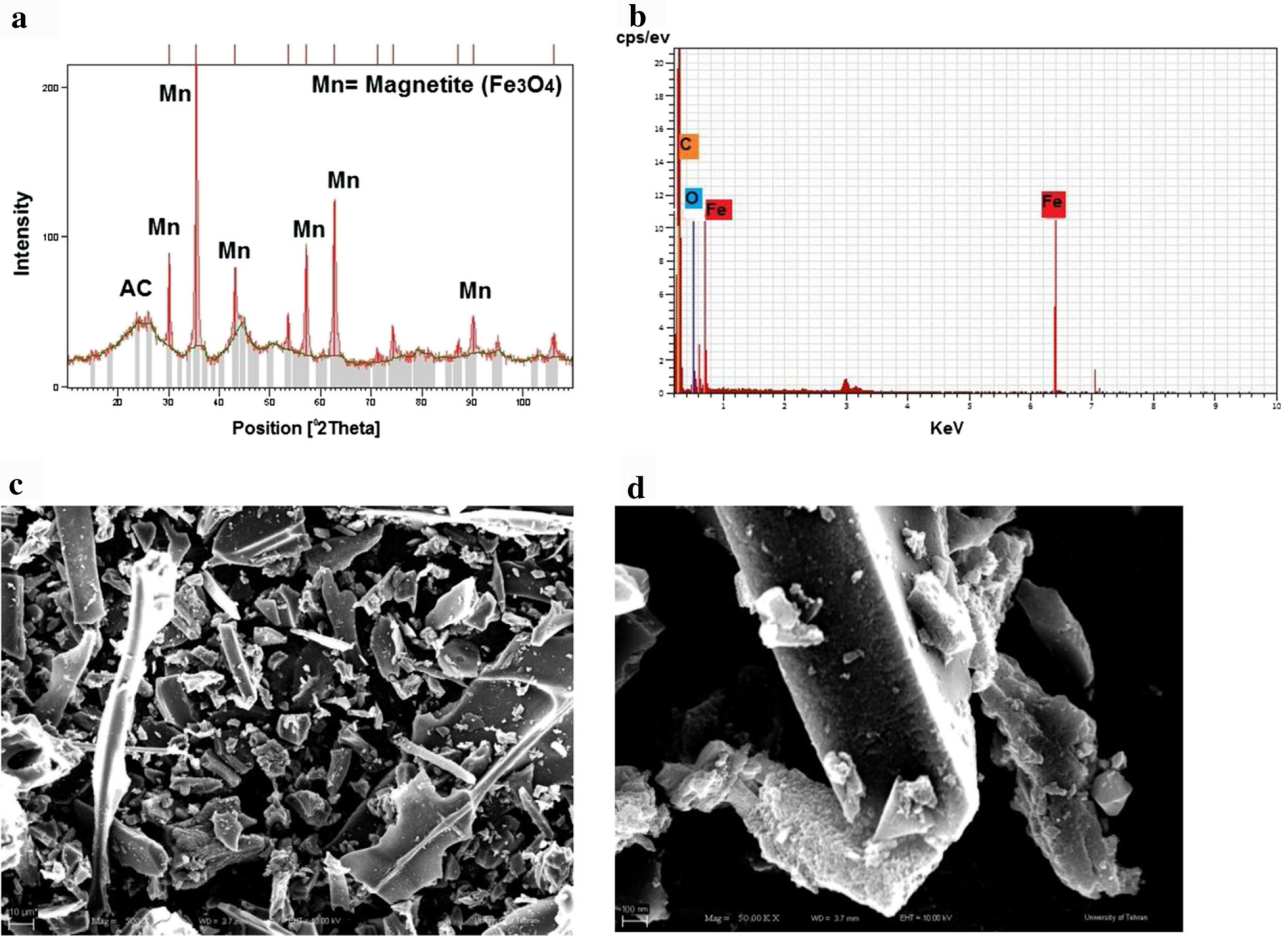
**Results of catalyst analysis. (a)** XRD pattern of the AC/nano-Fe_3_O_4_ composites; **(b)** Energy dispersive X-ray analysis spectrum of the nano-composite; **(c and d)** Typical SEM micrographs of sample (×500 and × 50000, respectively).

Figure 1a shows the X-ray diffraction (XRD) patterns of carbon nano-composite. Diffraction peaks assigned to the synthesized carbon nano-composites at 2θ = 24◦ indicating that the AC structure was not destroyed after the chemical calcination of the catalyst. Six characteristic peaks for Fe_3_O_4_ (2θ = 30.2°, 35.5°, 43.1°, 53.6°, 57.2°, 62.8°, 74.3° and 90.1°) were marked by their indices ((62), (188), (45), (22), (72), (102), (20.3) and (24.6)), observed for the carbon nano-composite, showing that the resulting magnetic nano-particles in the composite were actually pure Fe_3_O_4_. The result regarding the EDS analysis reveals mass fraction of elements of nano-composite, and showed that 89.20% of the synthesized catalyst contains pure carbon, while 5.04% and 5.76% of the total weight consists of iron and oxygen, respectively. No peaks of impurities were observed, indicating that the high purity catalyst was obtained. As can be seen in Figure 1b, Fe and O are the major elements of the coating.

Typical SEM micrographs of the catalyst are shown in Figure 1c and d. They represent the morphology of the nano-particles of Fe_3_O_4_ with the average particle sizes of 25–30 nm.

### Parameters affecting ozonation

#### Effect of pHzpc of the catalyst and initial pH of the solution

The catalyst surface will be charged negatively when pH > pHzpc, positively when pH < pHzpc and neutrally when pH ≈ pHzpc. pH of the solution can greatly affect the structural properties of the pollutant. Changes in the pH can alter the ions in solution, the ionic state of the phenol and the surface properties of the catalyst.

**Figure 2:**
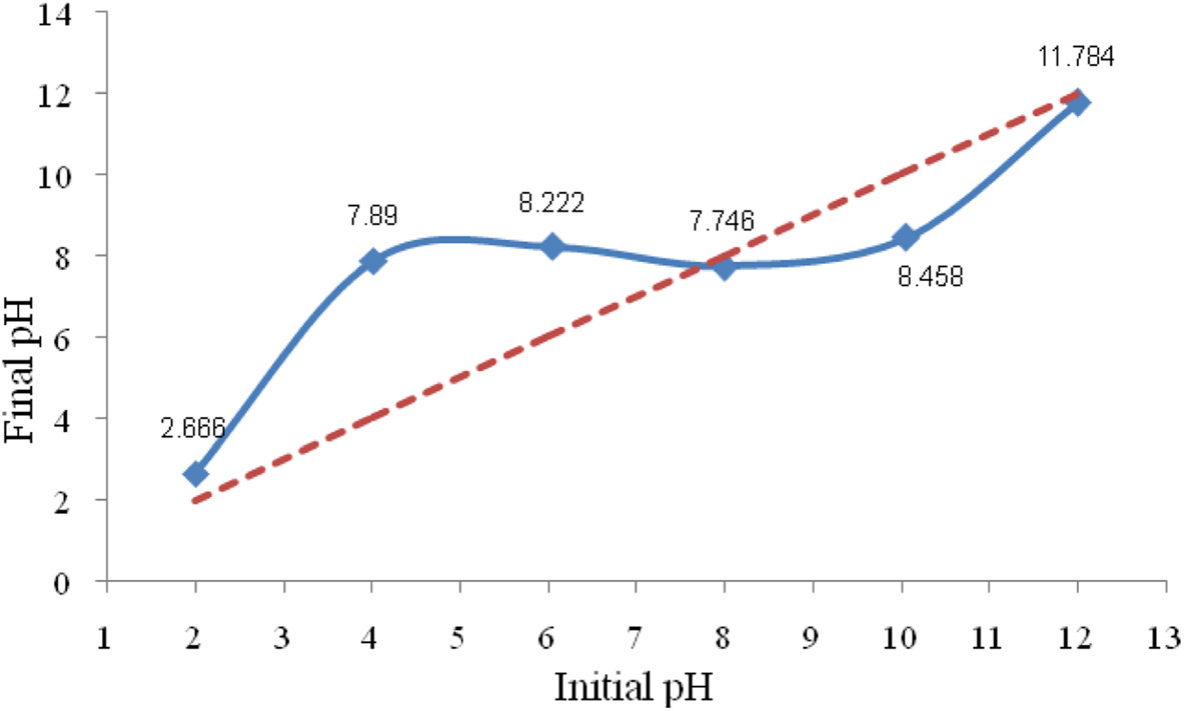
The pH change for determination of pHzpc of the catalyst.

The pHzpc of synthesized catalyst was measured 7.7 (Figure 2), falling in the range of 6.08–7.7, reported by various researchers for similar catalysts [11,24]. It is demonstrated that the catalyst surface has slightly basic properties. These basic functional groups located on the surface of the catalyst are thought to be responsible for ozone decomposition, resulting in the generation of reactive radical species [12].

Studies have shown that thermal modification of activated carbon under N2 stream could significant gasify oxygenated acid complexes such as carboxylic acid which formed in acid treatment. These surface oxygenated groups reduce electron density on carbon layers. Since ozone molecules are known to present electrophilic reaction with high electron density sites, and a reduction of carbon electron density should decrease the ozone molecules reactivity towards the carbon [25], this thermal treatment can improve the catalytic properties of the catalyst.

**Figure 3:**
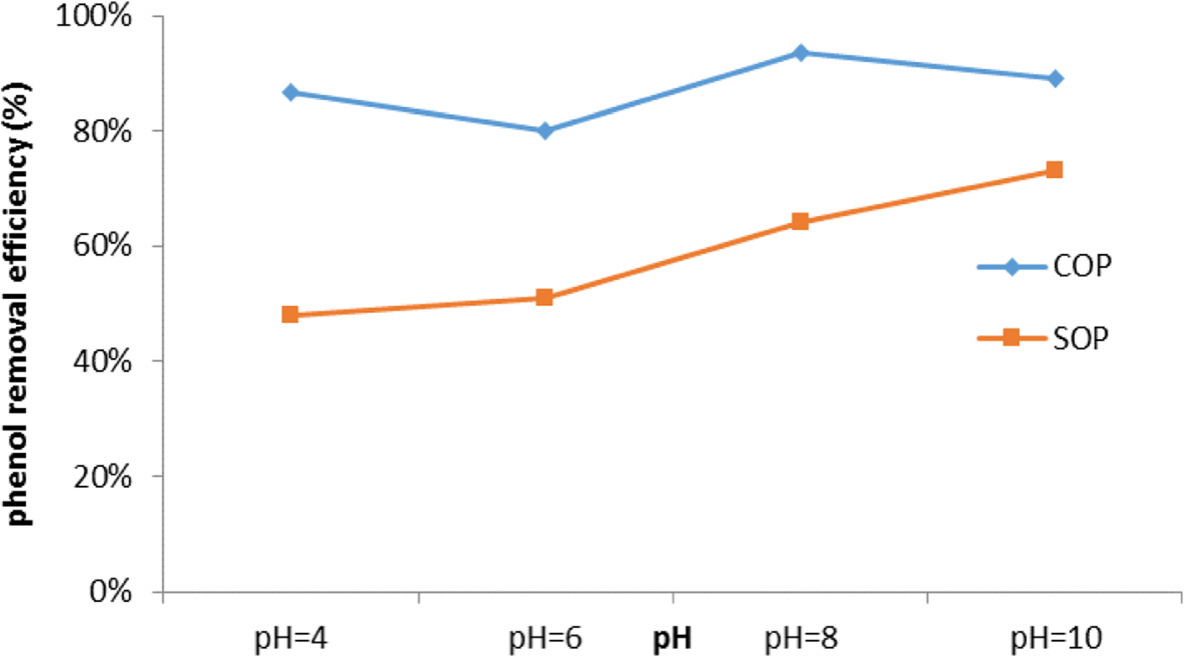
**Effect of initial pH on removal efficiency of phenol.** [Phenol]_0_: 100 mg/L; Catalyst concentration of 0.5 g/L; reaction time: 5 min.

As observed in Figure 3, the rate of phenol degradation in SOP increased from 48% at pH of 4 (almost linearly) to 73% at the pH of 10 during a 5-min reaction time. This increase can be attributed to the effect of pH value on the ozone transfer rate from the gas flow to the liquid phase [12] and increase the concentration of OH anions (decomposition of ozone to reactive oxidizing radical species with the much higher oxidation potential than ozone molecules in the solution) [26].

In COP, due to the properties of the catalyst surface, the maximum degradation (93%) was obtained at pH of 8 and upper than this pH (pH = 10), the degradation was decreased (89%) [4,27]. As shown, at higher pH, a negative effect on phenol degradation was observed, and the degradation rate of phenol was decreased under strong alkaline conditions (Figure 3). This result could be interpreted by considering both the property of phenol (pKa =9.9) and the surface nature of the catalyst, with respect to the pH of the solution and catalyst pHzpc.

Since the pHzpc of catalyst is 7.7, a negative charge is developed on its surface at this pH (>8), and since the pKa of phenol is 9.9, on the other hand, it is mostly dissociated to its ionic form (the phenolate anion) at above this pH value [28]. The effect of pH value on the adsorption of phenol can be explained by the electrostatic interaction between the surface of the catalyst and the target material and the affinity of catalyst toward phenolate through adsorption under strong alkaline condition is limited, leading to the reduction of phenol degradation rate [12].

We found out, as expected, that the optimal pH was in $pH_{\mathrm{ZPC}}^{\mathrm{catalyst}}<\mathrm{pH}<pK_{a}^{Ph\mathrm{enol}}$ range [26]. At this rang of pH the negatively charged catalyst and positively charged phenol molecules should readily attract each other [11].

Hence, under neutral and weak alkaline conditions (pH 7-8), negative charge is the predominant surface charge for the catalyst and the positive charges are the primary species of the phenol contaminant. Therefore, both of them are easily attracted toward each other through hydrogen bonding and consequently, the amount of phenol adsorption and its decomposition rate rise [27].

As Zhao et al. [16] pointed out, an increase was observed in the degradation rate of nitrobenzene in COP using a Mn catalyst with an increase in the pH of solution from 3 to 11.

In further developments, some researchers have found out that a decline in the mineralization of phenolic compounds, in a COP over Mn–Ce–O as a function of pH of solution between 3 and 10 [17].

It is also reported that the optimum pH for decolorization and mineralization of azo dye in COP using MgO nano-catalyst was found to be at alkaline pH over 8 [29].

It can be deduced that the process in which the pH of solution affects the degradation of a contaminant in COP, depends on the structure, the type of the reacting compound and the properties of the catalyst. Hence, the optimum pH of the COP must be selected for each specific condition.

Nonetheless, as shown in Table 3, the maximum degradation rate in the COP was obtained at pH of 8.0 and the phenol degradation rate was remarkably higher in the COP than that of the SOP, regardless of pH. It is reported that around 2.7 to 4.5-fold increase in the reaction rate constant will be observed, due to the fact that ozone reacts indirectly with organic molecules at alkaline pHs [18].

**Table 3:** Kinetic information of phenol removal as a function of pH in the SOP and COP

pH	4	6	8	10
COP				
Order	1	1	1	1
Constant (1/min)	0.425	0.2680	0.8750	0.7050
R^2^	0.98	0.97	0.92	0.96
SOP				
Order	1	1	1	1
Constant (1/min)	0.156	0.125	0.191	0.242
R^2^	0.98	0.98	0.98	0.97

### Effect of catalyst dosage

**Figure 4:**
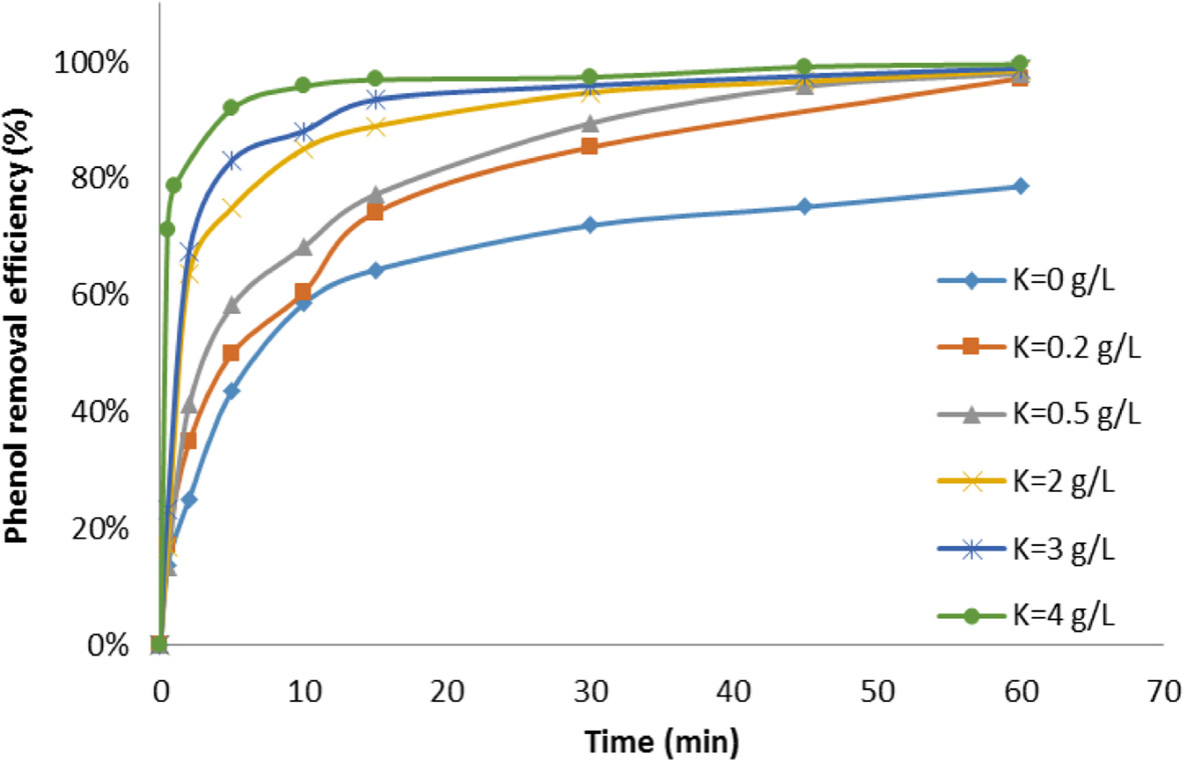
**Effect of catalyst concentration on phenol degradation efficiency in SOP and COP.** [Phenol]_0_: 500 mg/L; initial pH of 8.0.

As illustrated in phase 2 of Table 2, it can clearly be seen that the degradation of phenol, as a function of catalyst concentration (Figure 4), shows a significant enhancement effect of catalyst concentration on the ozonation process, particularly in the first minutes of the reaction time.

For example, at a given reaction time of 5 min the degradation of phenol increased from 43% in the absence of catalyst to 50% in the presence of 0.2 g/L solution of catalyst and then up to 92% at catalyst concentration of 4 g/L, representing the catalytic effect of catalyst concentration on ozonation followed by almost total removal of phenol (99%) once the reaction time was extended to 45 min. Table 4 shows the degradation rate of phenol at different concentrations of catalyst in the COP, compared to that of the SOP, representing similar removal sufficiency. For example, the reaction rate constant of phenol increased from 0.023 (1/min) in the SOP to 0.056 (1/min) in the case of COP (0.2 g/L of catalyst) and then to 0.071 (1/min) with catalyst concentration up to 4 g/L, indicating the 3-fold increase in the reaction rate constant in COP compared to that of SOP.

**Table 4:** Kinetic information of phenol degradation rate constant as a function of catalyst concentration in SOP and COP

**Catalyst concentration**	**k = 0 g/L**	**k = 0.2 g/ L**	**k = 0.5 g/ L**	**k = 2 g/ L**	**k = 3 g/ L**	**k = 4 g/ L**
K_1_ (1/min)	0.023	0.056	0.061	0.062	0.065	0.071
R^2^	0.81	0.98	0.98	0.89	0.85	0.81
lnC_0_	5.92	5.95	5.86	5.46	5.27	4.70

The increase of phenol degradation rate with an increase in catalyst concentration can be attributed to the expansion of surface area of the catalyst and the availability of more active sites for ozone decomposition leading to the enhancement of the following events:

The contact surface area for the reaction of phenol and ozone molecules [30], generation of reactive species of radicals [31-33] and the improvement of the phenol degradation efficiency.

Further increases in the catalyst concentration up to 4 g/L did not significantly affect the percentage of phenol degraded at the end of the reaction time (60 min). It is worth noting that the optimum concentration of the catalyst in COP strongly depends on the type of catalyst, the reaction conditions, the target compound and desired level of efficiency [11]. Hence, due to negligible increase in degradation efficiency by higher concentration of catalyst, in this study the value of 2 g/L was chosen as the optimum concentration for the catalyst (at initial concentration of phenol; 500 mg/L), and applied to further experiments.

The above-mentioned findings are in agreement with Qu et al. although different experimental conditions were applied in both methods [34].

### Mechanism of phenol degradation

Based on the literature, the most likely mechanism is as follows:

Ozone molecules are first adsorbed on the functional groups of the catalyst surface followed by decomposition by AC [4,7] and metal oxides including Fe_3_O_4_[35]. Afterwards, the generation of hydroxyl radicals and surface oxygenated radical species take place [36-38].

**Figure 5:**
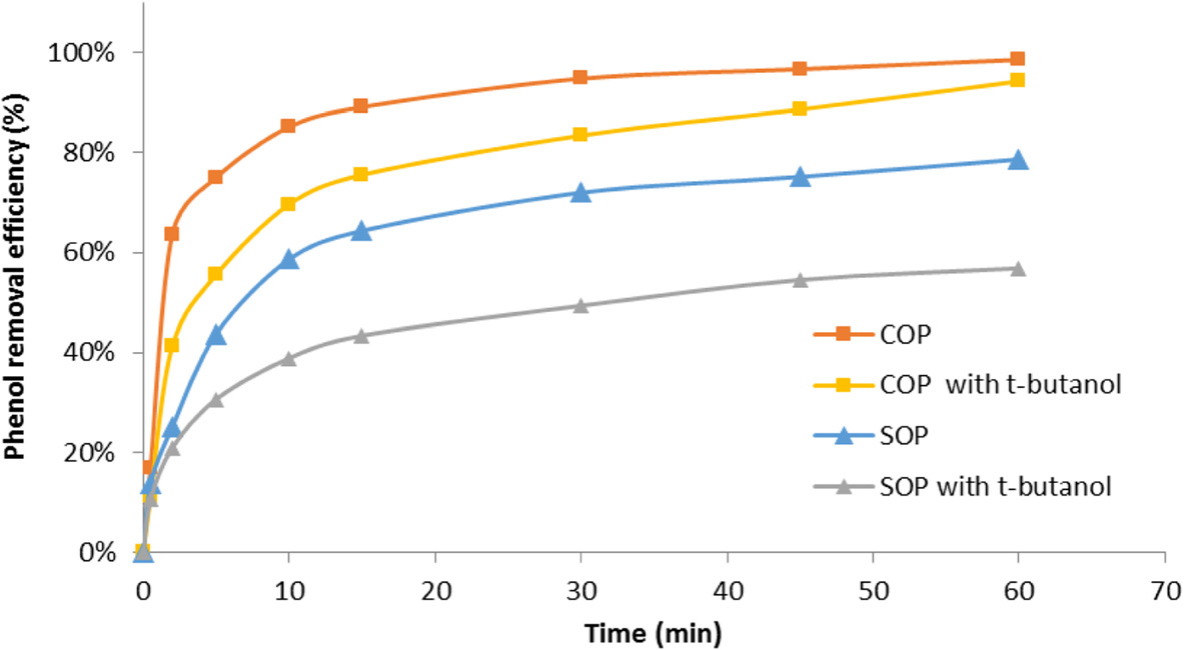
**Effect of t-buanol radical scavenger on phenol degradation in the SOP and COP.** [Phenol]_0_: 500 mg/L; Catalyst concentration of 2 g/L; initial pH of 8.0.

To evaluate the likelihood of the proposed mechanism, the degradation of phenol in COP was investigated by the radical scavenger experiment defined in phase 3 of Table 2.Figure 5 shows that the addition of t-butanol noticeably diminished the efficiency of phenol removal and the reaction rate constant in SOP from around 78% to 56% and 0.023 to 0.012 (1/min) respectively; while the corresponding figures in COP are from 98% to 94% and 0.062 to 0.042 (1/min), respectively.

In other words, the radical scavenger lowered the efficiency in SOP and COP (22% and 4%, respectively) and the reaction rate constant (48% and 32%, respectively), representing a 5.5-fold reduction in the degradation rate and 1.5-fold reduction in the reaction rate constant in SOP compared to those of COP.

Since •OH radicals are more reactive than ozone molecules [9,27] and t-butanol is a well-known •OH scavenger [39], indirect radical oxidation seems to be the predominant degradation mechanism in COP as observed in similar researches [17,40]; however, the high removal efficiency can be due to the fact of the capability of this catalyst to decompose ozone and thereby enhancement of reactive radicals generation [37,38]. As seen, the degradation of phenol in the COP in the presence of tert-butanol was slightly lower than that obtained in the absence of the scavenger under the same condition. The point that the degradation rate is not suppressed significantly by the presence of radical scavenger, especially at the end of reaction time, suggests that the surface of catalyst plays a key role in the oxidation of phenol [12] and other radical species specially •OH was the dominant species for oxidation process in the reactor [36].

This is an important part of this study because the radical scavengers that may be present in wastewaters will not interfere seriously with the degradation of the target contaminant.

To calculate the synergistic effect of the catalyst on the ozonation of phenol, the following equation is used [11,12]:

(2) $$\begin{array}{ll} \mathrm{Synergistic}\;\mathrm{effect}\;= & \;\mathrm{degradation}\;\mathrm{in}\;\mathrm{COP}\\ & \;-(\mathrm{degradation}\;\mathrm{in}\;\mathrm{SOP}\\ & \;+\mathrm{removal}\;\mathrm{by}\;\mathrm{catalyst}) \end{array}$$

**Figure 6:**
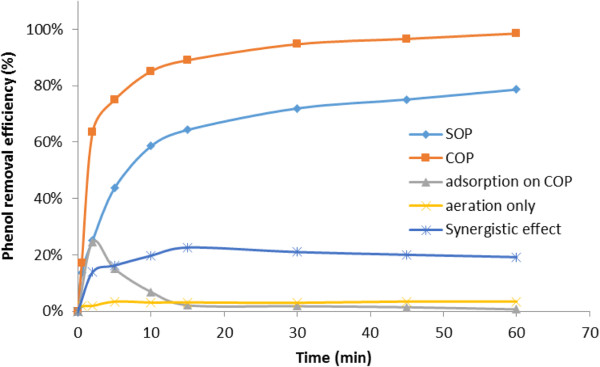
Synergistic effect of catalyst on ozonation process.

Results obtained from the experiments (Figure 6), as defined in phase 4 of Table 2, show that the catalytic role of magnetic carbon is not affected by adsorption [33], and also show a catalyst synergistic effect with ozonation degradation of phenol followed by decomposition of ozone, as observed by other researchers [27,41].

Some literatures have reported that the functional groups of sorbent surface can be modify by ozone, by increasing the specific surface area of the pores, and significant decreasing of the total volume of the catalyst [34], however, some others indicated that, once the activated carbon was ozonated, its specific surface area was slightly decreased, and the total volume of the pores remained unchanged [42]. Therefore, it can be concluded that the influence of ozonation on the structure of the carbon-based catalyst is dependent on its origin and nature.

### Mineralization of phenol in the COP and biodegradability of effluent

On completion of this investigation, the effect of the COP on the removal of COD from a phenolic sample was studied under the conditions defined in phase 5 of Table 2.

**Figure 7:**
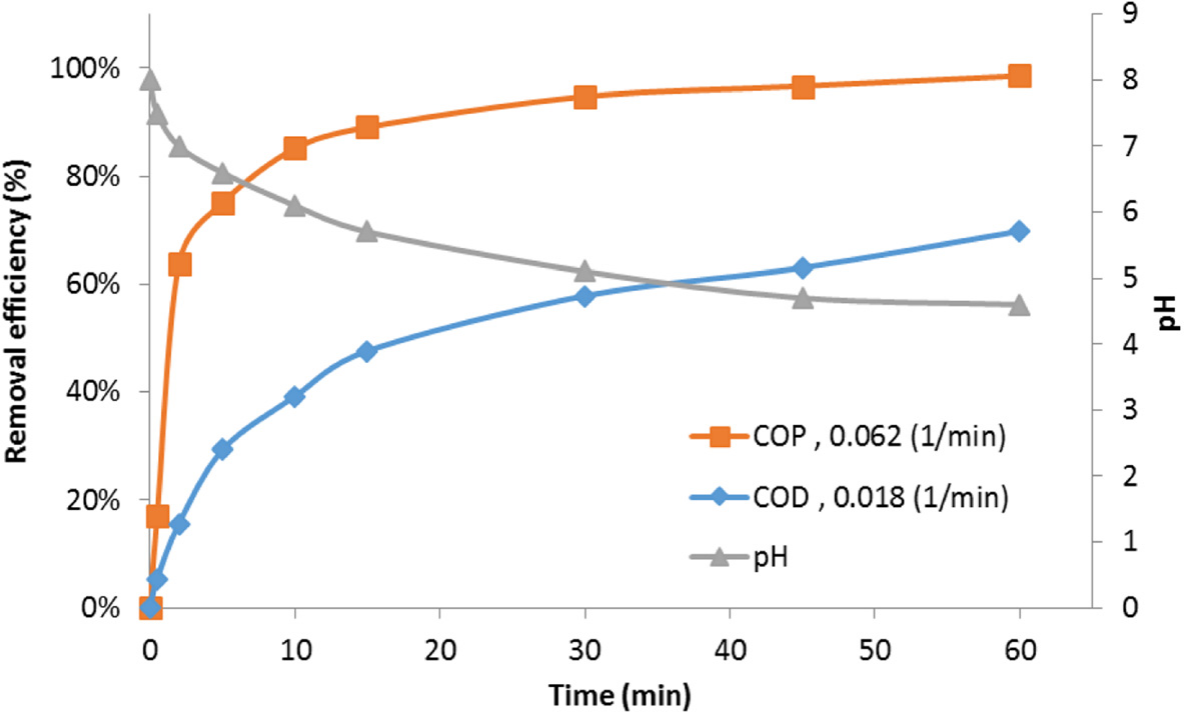
The degree of degradation and mineralization of phenol in the COP under optimal condition.

As reported in the literature, the AOP causes a drastic reduction of COD of recalcitrant organics [5]. Our findings indicate that the COP is not exempt from this general principle. As shown (Figure 7), the phenol degradation efficiency was approximately 85% after 10 min of the reaction. But, approximately 39% of the COD of phenol were removed after the same reaction time, and it increases up to 70% after 60 min; while in other similar studies, with other catalysts, COD removal efficiency from phenolic samples were lower than that the above mentioned values [43].

Although a further decrease (88%) was reported by Moussavi et al. [11] in the COP of phenolic sample (C_0_ = 1200 mg/L) at the end of reaction time (60 min), applying a 10-fold increase in the concentration of AC catalyst will overshadow its economic superiority. However, economical comparison of these catalysts needs more investigation.

It is also shown in Table 4 that the reaction rate constant for phenol degradation (0.062 1/min) decreased to one third of its initial level for COD removal (0.018 1/min).

The results show that the efficiency of COD removal and the reaction rate constants are lower than phenol degradation and its COD removal, particularly in early minutes of the reaction. This phenomenon can be illustrated in such a way that phenol molecules are converted to some intermediates prior to complete oxidation, especially at the initial steps of degradation.

According to some findings [11,43,44], major intermediates of phenol oxidation in AOP can be categorized as readily biodegradable compounds (acetic, fumaric, propionic, formic and succinic acids), non-biodegradable compounds but without inhibitory or toxic effect over the biomass (maleic, oxalic and malonic acids), toxic (p-benzoquinone and hydroquinone), and finally, inhibitory compounds (catechol) for the biodegradation [43,45].

The quick drop in pH value versus the reaction time confirms that acidic intermediates were generated during the oxidation of phenol (Figure 7). In addition, as the oxidation process is promoted by COP, the gap between COD and phenol removal percentage curves is narrowed. This could be due to the fact that intermediates are more efficiently mineralized in COP than their corresponding parent compounds. Overall, these results inferred that the COP with this catalyst could cause a high degree of degradation and mineralization of phenol compared to the other processes such as single ozonation [11,46], Fenton and photo-Fenton [47], Adsorption, TiO_2_-photocatalytic, wet air oxidation and catalytic-based wet air oxidation [48].

Although, the COP in the presence of nano-composite catalyst was able to degrade almost completely all the phenol (98.5%) and removed significant amounts of COD (70%) after 60 min of reaction time, the rather long time of ozonation would impose high operational cost for the generation of required ozone on the treatment system. Hence, the combination of the COP and SBR system features the benefits of both processes, shortening the reaction time required to achieve high effluent quality at the minimum cost.

**Figure 8:**
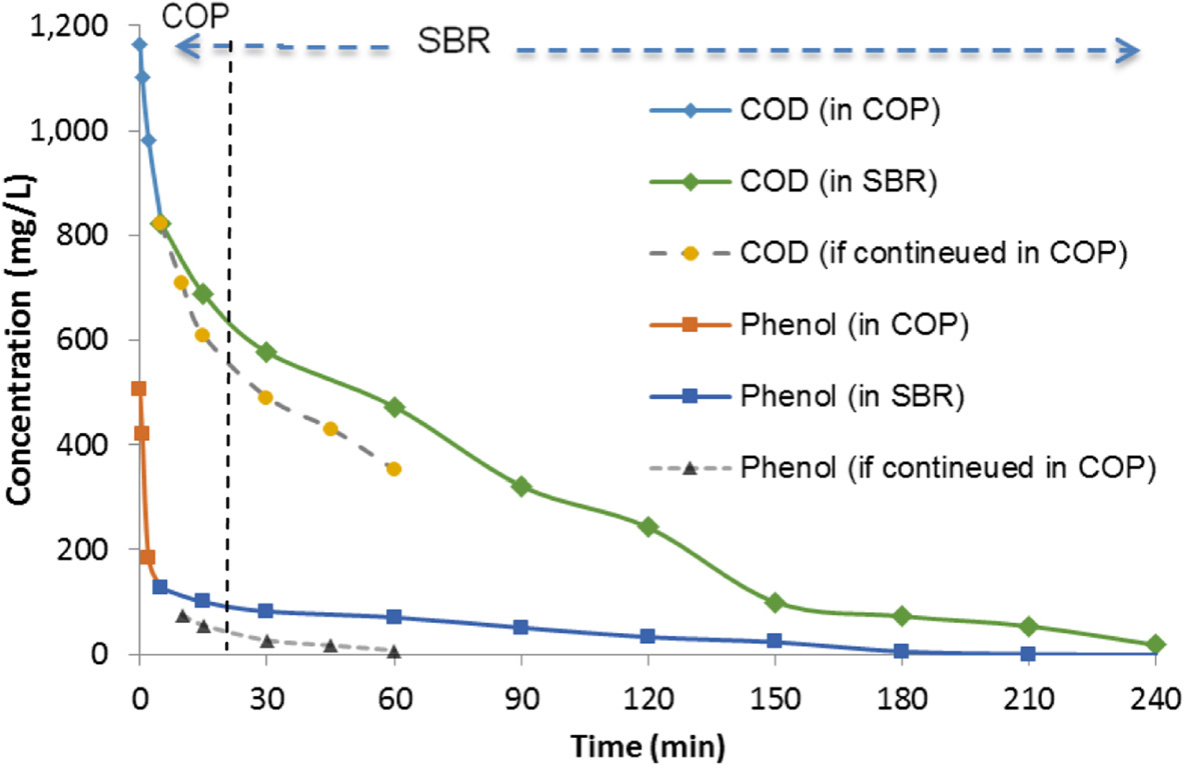
Biodegradability of COP effluent under optimal condition.

In this section, the effluent of COP obtained after 5 min of reaction time in COP under the optimal conditions, selected in Table 2- phase 6, was used for biological treatment. 5 min was chosen because at this time, the residual concentration of phenol and its associated COD had been reduced to below 126 and 822 mg/L, respectively (Figure 8), making that effluent suitable for biological treatment. The time evolution of phenol degradation and its associated COD concentration using the combined process of COP and biodegradation compared to those of single COP are demonstrated in Figure 8. As observed, the phenol and COD concentrations were reduced to zero and 18 mg/L after 4 h aeration in the combined system of the COP and SBR treatment, respectively.

The results also showed that the COP not only efficiently degraded the phenol as a recalcitrant compound, but also prompted a high degree of COD removal and consequently, the mineralization of its derivative intermediates. As other studies reported [46], catalytic ozonation process improved the biodegradability at BOD_5_/COD ratio of phenolic compounds from only 0.3 to 0.52 and slightly reduced toxicity of the intermediate solution. Similar results have also been achieved for biological treatment of non-biodegradable compounds following pre-oxidation with AOPs [27,29,34].

### Catalyst durability

From a practical point of view, the most important characteristics of the proposed catalyst are as follows: its minimal deactivation and the ability of being recovered by a magnet after the reaction.

**Figure 9:**
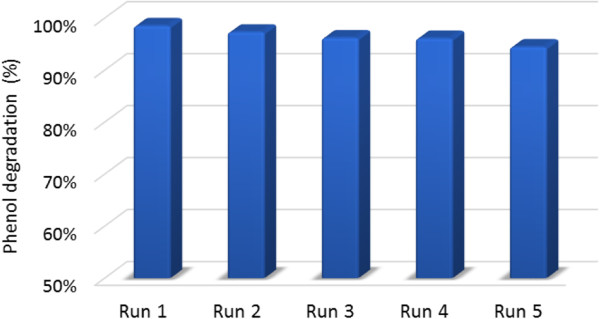
Effect of catalyst reuse on phenol degradation in the COP.

To evaluate the capability of the catalyst used in the COP after reuse, an experimental condition was defined (Table 2, phase 7). The results show (Figure 9) that this super-paramagnetic catalyst retained its catalytic and magnetic properties after 5-time reuse and the reduction in phenol removal was negligible (only 4%), which can be due to wash-out of catalysts from the system.

This can be illustrated either by the predominance of catalytic properties- rather than adsorption–oxidation processes- in the COP as mentioned before, or by the in situ regeneration of the catalyst [34]. Although, some researchers have reported that the contaminant removal has been improved after reuse, due to the modification of chemical functional groups on the catalyst surface by ozonation and the rise both in the pore volumes and the specific surface areas [49-51], others have indicated that ozonation reduces catalytic properties of activated carbon due to a decrease of basic groups and an increase in the number of oxygenated surface functional groups such as hydroxyl and carboxylic acid groups and nitro aromatic compounds [52].

## Conclusions

In this study, the preparation and the assessment of properties of AC/nano-Fe_3_O_4_ composite used as a catalyst in the catalytic ozonation of phenol have been investigated. This super paramagnetic nano-composite exhibited a catalytic effect on the ozone decomposition and reactive radical generation which was effectively separated from solution by applying a magnetic field and reused for several times. At the initial concentration of 500 mg/L of phenol and the optimal conditions as well as O_3_ dosage of 33 mg/(L · min), 98.5% and 69.8% of phenol and COD were removed in the COP, respectively. The findings indicated that phenol was mainly decomposed through a series of oxidation reactions occurring on the surface of the catalyst, and the radical scavengers present in wastewater could not affect the catalytic reaction. The capability of phenol and COD removal by combined system of COP and SBR was also investigated. The results clearly showed that the COP could improve the biodegradability and reduce the concentration of phenol and COD from around 500 and 1162 mg/L down to 126 and 822 mg/L after a few minutes, respectively. The effluent was then efficiently post-treated in an SBR bioreactor in which, at a relatively short aeration time the phenol was completely removed and COD was decreased to below 18 mg/L. Accordingly, it is concluded that this nano-composite is an efficient and active catalyst in the degradation and mineralization of phenol in the COP technique ,and combination of the biological process followed by COP is an effective and economic technique for the treatment of wastewaters containing recalcitrant contaminants, such as phenol.

## Competing interests

The authors declare that they have no competing interests.

## Authors’ contributions

All members of authors actively participated to carry out this investigation and had surveillance on this article. All authors read and approved the final manuscript.
